# Elephant-to-Human Transmission of Tuberculosis, 2009

**DOI:** 10.3201/eid1703101668

**Published:** 2011-03

**Authors:** Rendi Murphree, Jon V. Warkentin, John R. Dunn, William Schaffner, Timothy F. Jones

**Affiliations:** Author affiliations: Centers for Disease Control and Prevention, Atlanta, Georgia, USA (R. Murphree);; Tennessee Department of Health, Nashville, Tennessee, USA (R. Murphree, J.V. Warkentin, J.R. Dunn, T.F. Jones);; Vanderbilt University School of Medicine, Nashville (W. Schaffner, T.F. Jones)

**Keywords:** Mycobacterium tuberculosis, elephant, zoonoses, indirect transmission, tuberculosis and other mycobacteria, Tennessee, United States, research

## Abstract

In 2009, the Tennessee Department of Health received reports of 5 tuberculin skin test (TST) conversions among employees of an elephant refuge and isolation of *Mycobacterium tuberculosis* from a resident elephant. To determine the extent of the outbreak and identify risk factors for TST conversion, we conducted a cohort study and onsite assessment. Risk for conversion was increased for elephant caregivers and administrative employees working in the barn housing the *M. tuberculosis*–infected elephant or in offices connected to the barn (risk ratio 20.3, 95% confidence interval 2.8–146.7). Indirect exposure to aerosolized *M. tuberculosis* and delayed or inadequate infection control practices likely contributed to transmission. The following factors are needed to reduce risk for *M. tuberculosis* transmission in the captive elephant industry: increased knowledge about *M. tuberculosis* infection in elephants, improved infection control practices, and specific occupational health programs.

Zoonotic transmission of *Mycobacterium tuberculosis* from elephants to humans working in close proximity was described in the late 1990s. Studies of workers exposed to elephants infected with *M. tuberculosis* have reported a potential for elevated risk among those who have prolonged and close contact with elephants; engage in treatment, medical procedures, or necropsies of elephants; live inside or close to an elephant barn; or participate in cleaning elephant barns or work as groundskeepers (*1*–*3*).

In North America, ≈270 Asian and ≈220 African elephants live in captivity (*4*,*5*), most in facilities accredited by the Association of Zoos and Aquariums and the rest in public, private, and nonprofit facilities. Among these, ≈12% of Asian and ≈2% of African elephants are thought to be infected with *M. tuberculosis* (*6*,*7*). Recommendations for detection and treatment of tuberculosis (TB) in elephants exist (*8*). However, no standard definition exists for latent TB in elephants, and no sound clinical criteria exist for diagnosing TB in elephants. Elephants are considered exposed to *M. tuberculosis* if they have had contact with an *M. tuberculosis* culture–positive animal. They are thought to have active TB when *M. tuberculosis* is cultured from respiratory secretions obtained from their trunk (trunk wash). However, performing a trunk wash is challenging, and culture of *M. tuberculosis* from these specimens is unreliable (*9*,*10*). Knowledge about effectiveness of human antituberculous medications in elephants is limited (*6*–*8*).

We describe an outbreak of *M. tuberculosis* infection among employees of an elephant refuge. We also present findings of the ensuing epidemiologic and environmental investigation conducted to identify work practices and facility characteristics that probably contributed to zoonotic transmission.

## Outbreak

In July 2009, routine screening detected conversion of tuberculin skin test (TST) results from negative to positive among caregivers at a nonprofit elephant refuge in south-central Tennessee, USA. In addition, records review revealed that respiratory secretions obtained by trunk wash of a quarantined elephant (elephant L) in December 2008 contained *M. tuberculosis*. To determine the extent of the outbreak, identify risk factors for TST conversion among humans, and develop strategies to prevent ongoing zoonotic transmission, we conducted an investigation.

## Setting

The elephant refuge was established in 1995 with the mission of caring for sick, old, or abused elephants transferred from private owners, zoos, and circuses. It operates on 2,700 acres divided by fences into 3 distinct areas, each having its own barn. Elephants graze outdoors during the day and might be enclosed in barns at night, particularly during cold or inclement weather. At the time of the outbreak, 1 area housed 2 African elephants; 1 area housed 6 Asian elephants; and a third area housed 7 Asian elephants in a large quarantine barn connected to a 2-story administrative support building. The refuge is accredited by the Association of Sanctuaries and licensed by the United States Department of Agriculture (USDA) and the Tennessee Wildlife Resources Agency (TWRA); it is closed to the public.

In 2004, the refuge received, from an exotic animal farm in Illinois, 2 female Asian elephants with a history of active TB. The transfer of *M. tuberculosis* culture–positive elephants into Tennessee was contingent upon adherence to the USDA-endorsed Guidelines for the Control of Tuberculosis in Elephants (*8*) and an infection control plan set forth by TWRA in consultation with the Tennessee Department of Health (TDH). One elephant died of TB in 2005; the other was treated with antituberculous medications for 1 year and was released from isolation in accordance with the guidelines.

In 2006, the refuge accepted 8 additional elephants from the same exotic animal farm in Illinois. Although none were known to have active TB, they were considered exposed and at high risk for latent *M. tuberculosis* infection because they had been housed with *M. tuberculosis* culture–positive elephants. In accordance with the guidelines, all 8 were quarantined when they arrived in Tennessee, and respiratory secretions obtained by trunk wash were tested annually for *M. tuberculosis*. In 2008, one died of causes unrelated to TB.

## Investigation

Information and records provided by the refuge and TWRA were used to construct a historical timeline of key events for employees and resident elephants. Onsite evaluations of barn management and husbandry practices were conducted.

Elephants at the refuge had been trained to give respiratory secretions that were used for culture isolation of *M. tuberculosis* by a triple-sample trunk-wash method (*8*). Briefly, 30–60 mL of sterile saline was instilled into the elephant’s trunk. The elephant raised and then lowered its trunk to drain or exhale the saline into a plastic bag. Three samples obtained on separate mornings within 1 week were processed by using standard methods for culture isolation of mycobacteria (*11*). *M. tuberculosis* isolates obtained from elephant respiratory secretions were genotyped by using standard methods recommended by the Centers for Disease Control and Prevention (CDC). Results were compared with others stored in the CDC TB Genotyping Information Management System.

A retrospective cohort study was conducted to identify risk factors for *M. tuberculosis* infection among employees who worked at the elephant refuge during 2006–2009. One investigator interviewed current employees in person and former employees by telephone. Employees were asked about potential risk factors for *M. tuberculosis* exposure, history of TSTs and *M. tuberculosis* infection, work assignments and practices, training and use of personal protective equipment, and close contact with elephants. Close contact was defined as touching or being close enough to touch an elephant. TST results, employment history, and N95 respirator fit-testing dates were verified by checking employee records at the refuge.

A preemployment TST was required for elephant caregivers, and all employees received annual TST screening. For employees with a documented negative (<10 mm) TST result, a >10 mm increase in induration within 2 years was considered a TST conversion indicative of recent infection with *M. tuberculosis* (*12*–*14*). Employees whose TST results converted were evaluated for latent *M. tuberculosis* infection or active TB at local health departments or by private clinicians.

Environmental samples were collected from the barn housing the *M. tuberculosis*–infected elephant. These included elephant feces (triplicate samples of 10 g each), water from drinking troughs (triplicate samples of 45 mL each), and swabs of barn surfaces (e.g., duplicate or triplicate samples collected from walls, floors, gates, and drains by using a 3M Sponge-Stick with neutralizing buffer [3M, St. Paul, MN, USA]). Samples were processed by TDH Laboratory Services according to standard methods for culture isolation of mycobacteria (*11*). A theatrical smoke machine was used to enable visualization of air flow patterns within and between the barn and administrative support areas.

Statistical analyses were performed by using SAS version 9.1 (SAS Institute Inc., Cary, NC, USA). CDC human subjects review classified this work as public health evaluation and control.

## Findings

All trunk-wash specimens obtained from elephants at the refuge during 2006–2009 had negative *M. tuberculosis* culture results except for specimens taken in December 2008 from 1 elephant living in the quarantine area (elephant L). *M. tuberculosis* culture–positive results were received in March 2009. In July 2009, sampling was repeated and culture-positive results were confirmed. Infection control practices were heightened in October 2009. In 2010, treatment of elephant L with antituberculous medication began but was complicated by the elephant’s intolerance to both oral and rectal medical therapy.

*M. tuberculosis* isolated from elephant L was susceptible to isoniazid, rifampin, ethambutol, pyrazinamide, and streptomycin and had genotype PCR01621. TDH records indicated that genotype PCR01621 had also been isolated from 2 elephants that had died with TB at the refuge in 2005 and 2006. All 3 elephants had spent time at the same exotic animal farm in Illinois, and at least 1 was among the elephants involved in a 1996 outbreak (*2*). *M. tuberculosis* PCR01621 was also isolated from an elephant in Missouri in 2008 and from a human patient who received a diagnosis of active TB in 2004. The human patient lived in California at the time of diagnosis; his potential for exposure to captive elephants was unknown.

Of 57 refuge employees, 46 (81%) were contacted (25 current and 21 former employees). Interviews were conducted with these 30 caregivers, 11 administrators, and 5 maintenance workers. Eleven former employees could not be reached. The average age of respondents was 38 years (range 20–65 years); 31 (67%) were female. All had at least 1 previous negative TST result; 9 had TST results that converted during 2006–2009 (indurations 12–24 mm), although none were identified as having active TB.

Relative risk estimates for traditional risk factors for TST conversion among refuge employees were not statistically significant (Table 1). No human source of *M. tuberculosis* at the refuge was identified. However, employees who worked in the quarantine area during 2009 were significantly more likely than those who did not work there during that period to convert (risk ratio 20.3; 95% confidence interval 2.8–146.7). One employee converted in 2006 after close, prolonged contact with the elephant that died with TB in 2005. The other 8 converted during 2009 and had worked in the quarantine area for >4 hours that year. Therefore, we separately examined characteristics of all 13 employees who worked in the quarantine area for >4 hours during 2009 and observed their work practices in more detail (Figure).

**Table 1:** Exact relative risk for potential risk factors for *Mycobacterium tuberculosis* infection among 46 elephant refuge employees, Tennessee, USA, 2009*

Potential risk factor	TST conversion/ risk factor, no. (%)	TST conversion/ no risk factor, no. (%)	Relative risk (95% CI)†
Foreign born	2/6 (33)	7/40 (18)	1.91 (0.51–7.10)
International travel past 5 y	5/19 (26)	4/27 (14)	1.78 (0.58–5.76)
Exposure to person(s) with TB	0/4 (0)	9/42 (21)	NC
Previous health care facility work	1/8 (13)	8/38 (21)	0.59 (0.09–4.10)
Previous correctional facility work	0/4 (0)	9/42 (21)	NC
Previous homeless shelter work	0/1 (0)	9/45 (20)	NC
Close contact with elephant(s)	2/11 (18)	7/35 (20)	0.91 (0.22–3.75)
Quarantine area exposure during 2009	8/13 (62)	1/33 (3)	20.31 (2.81–146.69)

*TST, tuberculin skin test; CI, confidence interval; TB, tuberculosis; NC, not computed. †Relative risk and confidence intervals were not computed when at least 1 cell contained zero.

**Figure:**
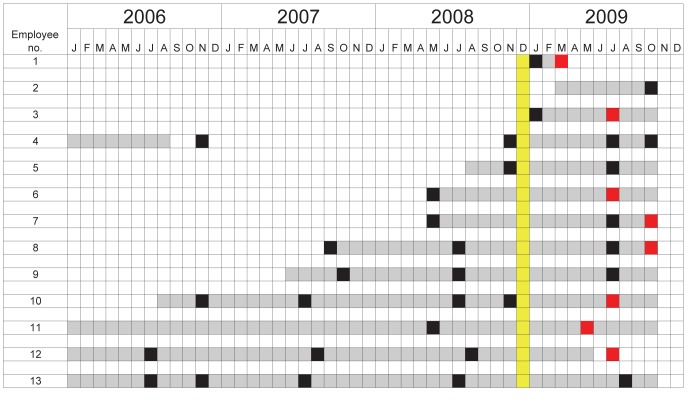
Tuberculin skin test (TST) conversion timeline for 13 employees who worked in the quarantine area of an elephant refuge, Tennessee, USA, 2009. Gray, exposure to quarantine barn; black, negative TST result; red, positive TST result; yellow, elephant L positive for *Mycobacterium tuberculosis*.

Among these 13 employees, only 1 who converted had close contact with any elephant (Table 2). Compared with employees who did not convert, fewer employees who converted reported always wearing an N95 respirator when indicated or having the fit of the respirator tested annually.

**Table 2:** Exact relative risk for potential risk factors for latent *Mycobacterium tuberculosis* infection among 13 employees who worked in the quarantine area of an elephant refuge, Tennessee, USA, 2009*

Potential risk factor	TST conversion/ risk factor, no. (%)	TST conversion/ no risk factor, no. (%)	Relative risk (95% CI)†
Close contact with elephant(s)	1/3 (33)	7/10 (70)	0.48 (0.09–2.48)
Participated in elephant trunk washes	0/1 (0)	8/12 (67)	NC
Pressure washed barn walls and floors	5/8 (63)	3/5 (60)	1.04 (0.43–2.55)
N95 respirator fit tested annually	2/5 (40)	6/8 (75)	0.53 (0.17–1.68)
“Always” compliant with N95 wear	2/5 (40)	6/8 (75)	0.53 (0.17–1.68)

*TST, tuberculin skin test; CI, confidence interval; NC, not computed. †Relative risk and confidence intervals were not computed when at least 1 cell contained zero.

Also among these 13 employees, 5 were elephant caregivers, 2 were maintenance workers, and 3 were administrators. Caregivers and maintenance workers engaged in aerosol-generating work practices during quarantine barn maintenance. Hay, sawdust, and excrement were swept or shoveled from elephant stalls and yards every day. In addition, the entire barn was cleaned every day with a high-pressure water sprayer. This practice created a dense mist that visibly lingered in the enclosed barn for hours. Although respirators were indicated for persons working inside the quarantine barn, construction of the barn allowed unfiltered air to flow between the barn and the adjacent 2-story administrative support areas where respirators were not worn. These administrative areas included space for barn storage, elephant food preparation, and data entry; a restroom on the first floor; and an office on the second floor. All 3 administrators who worked in these areas had no direct contact with elephants, but their TST results converted.

*M. tuberculosis* was not isolated from 52 samples collected from the environment of elephant L. *M. fortuitum* complex was identified in fecal samples from 2 elephants, a water sample, and surface swabs of a watering trough and 2 barn drains. Studies using theatrical smoke confirmed that air was shared between the barn and the 2-story administrative support area under normal working conditions.

The refuge infection control plan was intended for implementation when an *M. tuberculosis* culture–positive elephant was in residence. Before arrival of the 2 culture-positive elephants in 2004, employees had received training on the risk for *M. tuberculosis* transmission from elephants to humans, and the refuge established respiratory protection and TST screening programs. By the end of 2005, all elephants living at the refuge were *M. tuberculosis* culture negative. Subsequently, formal employee training was discontinued, and infection control procedures were not strictly followed. The refuge continued single preemployment TST screening for elephant caregivers and annual TST screening for all employees.

## Discussion

Epidemiologic and observational data indicate that *M. tuberculosis* was transmitted from an elephant with active TB to humans working at the elephant refuge. Employees who worked >4 hours in the quarantine barn during 2009 were 20× more likely to have latent *M. tuberculosis* infection than those who did not. TST results for refuge employees without quarantine barn exposure in 2009 did not convert. Risk for employees working in the quarantine area was probably increased by delayed response and failure to enhance infection control practices after obtaining *M. tuberculosis* culture–positive results for elephant L. Notably, close contact with elephant L was not required for transmission. Caregivers and maintenance workers probably aerosolized *M. tuberculosis* that had been expelled or excreted by elephant L while they cleaned soiled barn surfaces. For example, pressure washing created an impressively dense mist that lingered in the barn throughout the day. The mist was not contained within the barn and mixed with air in the connected administrative support areas where respirators were never worn, thus providing a route of indirect *M. tuberculosis* transmission for the 3 administrative workers who reported no contact with elephant L. The hypothesis of indirect transmission is further supported by the TST conversion of a study investigator who spent limited time in the administrative support area before the risk was recognized and interventions were implemented.

During the 19th and 20th centuries, disease caused by *M. tuberculosis* among captive elephants living in Asia, Europe, and North America was sporadically reported (*14*–*19*). The first reported outbreak of TB among elephants in North America occurred at an exotic animal farm in Illinois in 1996 (*2*). The investigation identified evidence of *M. tuberculosis* infection in 4 Asian elephants (3 of which died) and 11 elephant caregivers (1 of whom had active TB). The event prompted action from USDA, and since 1998, the USDA Animal Plant Health Inspection Service has required annual *M. tuberculosis* testing by the trunk-wash–culture method for all captive elephants in the United States (*20*).

Analogous to culture-positive sputum in human patients, an *M. tuberculosis* culture-positive trunk wash from an elephant is considered the standard for confirming active TB disease. However, active TB disease and shedding of *M. tuberculosis* organisms cannot be excluded with a culture-negative trunk-wash result because the test has low sensitivity (*9*,*10*,*21*).

Considerable effort has gone toward developing methods for early and reliable diagnosis of latent *M. tuberculosis* infection among elephants. TST is unreliable (*10*), but serum antibody tests appear promising (*9*,*22*). Although serologic tests can detect infection with *M. tuberculosis* years earlier than trunk-wash cultures (*9*), negative serologic results cannot exclude the possibility of infection. In February 2010, USDA added serologic testing (ElephantTB STAT PAK Kit; Chembio Diagnostic Systems, Inc., Medford, NY, USA) to its annual trunk-wash culture requirement for all elephants (*8*).

Knowledge gaps exist about the timing between elephant exposure, seroconversion, latent infection, active disease, and shedding. To improve medical management of elephants and to reduce the risk for transmission to other animals and humans, a better understanding of *M. tuberculosis* infection among elephants is crucial. Gaps also exist in knowledge regarding treatment and cure of elephants with *M. tuberculosis* infection. Although antituberculous medications used to treat humans are thought to be effective for treating active TB in elephants, little evidence is available to guide decisions regarding medication choice, dosage, length of treatment, or assessment for cure. Also unclear is whether treatment of elephants with *M. tuberculosis* infection successfully prevents progression to active TB disease.

Our findings highlight the effects of gaps in scientific knowledge and provide new information on potential risk factors for zoonotic transmission of *M. tuberculosis*. First, in this outbreak the inability to accurately and expeditiously detect *M. tuberculosis* infection and disease in elephants contributed to unrecognized, and therefore uncontrolled, risk. Improved methods for diagnosis of *M. tuberculosis* infection in elephants are needed. Second, infection control practices were insufficient to protect employees, creating an argument for detailed evidence-based guidelines and a more comprehensive approach to implementation. Third, employees were largely unaware of the risk for zoonotic *M. tuberculosis* transmission and the need to use adequate respiratory protection. Because risk cannot be eliminated, a strong occupational health and training program is needed for employees who work in facilities that house elephants potentially exposed to *M. tuberculosis*. Finally, our study suggests that employees without close contact with elephant L were infected through indirect transmission of *M. tuberculosis* aerosolized during routine barn maintenance (i.e., pressure washing or sweeping waste) or suspended in shared air. Reasonable efforts to reduce aerosol-generating practices and to limit aerosol spread in this unique environment should be considered.

The One Health movement argues for integrating human and veterinary medicine to defend the health and well-being of all animal species (*23*). This report provides a textbook illustration of this need. Captive elephants have emerged as an unanticipated source of *M. tuberculosis* infection among humans and therefore must be integrated in our strategies to control and eliminate TB. Because of the gaps in scientific knowledge, the high prevalence of *M. tuberculosis* infection among elephants living in North America, and the insensitivity of diagnostic tests, a substantial need exists for focusing attention on infection control practices and occupational health programs specifically designed to reduce zoonotic *M. tuberculosis* transmission in the captive elephant industry.
